# The Photoinitiators Used in Resin Based Dental Composite—A Review and Future Perspectives

**DOI:** 10.3390/polym13030470

**Published:** 2021-02-02

**Authors:** Andrea Kowalska, Jerzy Sokolowski, Kinga Bociong

**Affiliations:** Department of General Dentistry, Medical University of Lodz, 92-213 Lodz, Poland; andrea.kowalska@stud.umed.lodz.pl (A.K.); jerzy.sokolowski@umed.lodz.pl (J.S.)

**Keywords:** resin-based composite, photoinitiator, alternative photoinitiators, restorative dentistry, dental composites, camphorquinone, 1-phenyl-1,2-propanodione, trimethylbenzoyl-diphenylphosphine oxide, benzoyl peroxide

## Abstract

The presented paper concerns current knowledge of commercial and alternative photoinitiator systems used in dentistry. It discusses alternative and commercial photoinitiators and focuses on mechanisms of polymerization process, in vitro measurement methods and factors influencing the degree of conversion and hardness of dental resins. PubMed, Academia.edu, Google Scholar, Elsevier, ResearchGate and Mendeley, analysis from 1985 to 2020 were searched electronically with appropriate keywords. Over 60 articles were chosen based on relevance to this review. Dental light-cured composites are the most common filling used in dentistry, but every photoinitiator system requires proper light-curing system with suitable spectrum of light. Alternation of photoinitiator might cause changing the values of biomechanical properties such as: degree of conversion, hardness, biocompatibility. This review contains comparison of biomechanical properties of dental composites including different photosensitizers among other: camphorquinone, phenanthrenequinone, benzophenone and 1-phenyl-1,2 propanedione, trimethylbenzoyl-diphenylphosphine oxide, benzoyl peroxide. The major aim of this article was to point out alternative photoinitiators which would compensate the disadvantages of camphorquinone such as: yellow staining or poor biocompatibility and also would have mechanical properties as satisfactory as camphorquinone. Research showed there is not an adequate photoinitiator which can be as sufficient as camphorquinone (CQ), but alternative photosensitizers like: benzoyl germanium or novel acylphosphine oxide photoinitiators used synergistically with CQ are able to improve aesthetic properties and degree of conversion of dental resin.

## 1. Introduction

Light-cured dental composites changed old poor dentistry into a modern and esthetic branch of medicine. This process had begun in 1955 when Michael Buonocore discovered a simple method of increasing the adhesion of acrylic fillings to enamel by using orthophosphoric acid [1]. The second step was Dr. Bowen’s discovery: bisphenol A-glycidyl methacrylate (Bis-GMA resin), which has been a basic compound of dental composites since 1960. In 1975 for the first time dental resin composite was cured by light [2]. The change of curing dental composites eliminated the porosity of the composite and let the dentist control application of material into dental cavity. Dentistry took a big step that day, but there was still progress to make.

In recent years, the components of dental composites have been changing in order to improve the biomechanical and chemical properties. The properties of dental composites are divided into three groups. It is caused by compounds of the dental resins. The first group includes strength, stiffness, abrasion resistance and coefficient of thermal expansion. All three components of the composite: filler, matrix and coupling agent, are responsible for these features. The softening tendency and color stability are in the second group and it is caused by the type of matrix and photoinitiator. The last group contains polymerization shrinkage and water sorption. The last group is connected mostly with the type of matrix [3].

The current dental composites are composed of: organic resin matrix, inorganic fillers, coupling agent [1,3]. The most common monomers used for the matrix are: bisphenol A (Bis-GMA), ethoxylated bisphenol a glycol dimethacrylate (Bis-EMA), triethylene glycol dimethacrylate (TEGDMA), hydroxyethyl methacrylate (HEMA) and urethane dimethacrylate (UDMA) [2]. The others ingredients of matrix are: initiator system for free radical polymerization and stabilizers for maximizing the storage stability [3]. The inorganic fillers are responsible for mechanical properties especially for strength and abrasion resistance of the material. The fillers can be classified on the size of the particles: macrofill, microfill, hybrid, minifill, microhybrid, nanofill and the range of size of filler particles is from 5 nm to 50 µm [2]. This classification was made by Lutz and Phillips, but it did not originally contain nanofills because it was invented later. Willems et al. in 1993 created a more detailed classification which is based on parameters such as Young’s modulus, size of the main particles, surface roughness etc. Nanotechnology allowed dental manufacturers to produce high loaded composite up to 79.5%. These nanoparticles give better finish, the fillings are smoother, the material’s biodegradation over time is reduced and they also reduce curing shrinkage, marginal leakage, color changes and bacterial penetration [1]. Mostly fused silica, glass, quartz are used in dental composites as fillers [3]. The third important component of dental composite is coupling agent. The coupling agent is bonding inorganic fillers with organic matrix. There are three common coupling agents: zirconate, titanate and silanes [2]. Sometimes, fluorescent agents are added to improve optical aspect of composite to look as natural as dental tissue. The additives manage to cover yellow color of composites, by the reflecting amount of blue light. These are dyes or pigments that absorb light in the ultraviolet and violet region (340–370 nm) [4].

The common dental composites are cured by the light activation. It is possible due to presence of the initiator system of photopolymerization and its amounts varying from 0.1% to 1 wt% [4,5]. The amount of the initiator depends on the type of photosensitizer. The optimal concentrations of initiator in resin-based composites depend on many factors such as solubility of these compounds in the monomer, photoreactivity, color or biocompatibility [5].

The first aim of this review is to find an alternative photoinitiator which would compensate the disadvantages of camphorquinone, such as yellow staining or poor biocompatibility, and also would have mechanical properties as satisfactory as camphorquinone. The second goal is to assess the alteration of properties of dental resins when camphorquinone is used synergistically with alternative photoinitiators.

## 2. Materials and Methods

The main source of this review of literature was PubMed. Other sources were: Academia.edu, Google Scholar, Elsevier, ResearchGate and Mendeley. Many studies were manually found from references from relevant articles. Keywords used during searching: dental photoinitiator, resin-based composite, composite, polymerization, type-1 photoinitiators, type-2 photoinitiators, CQ, camphorquinone, benzophenone, PPD, 1-phenyl-1,2 propanedione, BAPO, TPO, Lucirin TPO, Ivocerin, phosphine oxide, alternative photoinitiator, unconventional photoinitiator. Over 6000 studies were found, and after scrutiny of searching, 1000 records were specifically checked. The 64 articles were selected to create this review.

Criteria for selection: the aim of this review was to present the most promising photoinitiators which can be use in future in dental industry. Studies were selected on relevance containing recent information about properties of dental composites including different photosensitizers.

## 3. Polymerization of Resin Matrix

Polymerization is a process when monomers react together to convert into polymers. The monomers used in dentistry: Bis-GMA, TEGDMA, UDMA etc., are liquids and as a result of polymerization they become solids [2]. The process of polymerization has three phases: initiation, propagation and termination. Free radicals are necessary to lengthen the chain of polymer and they are formed by photoinitiators [1]. Free radicals can be produced by a variety of thermal, photochemical and redox methods [5]. Hence, dental composites can be light-cured, chemo-cured or dual-cured [6].

Chemical activation is a reaction between an organic amine—catalyst paste with an organic peroxide—universal paste. After mixing these two pastes free radicals are produced. The free radicals attack the carbon double bonds and the process of polymerization begins. This process runs rapidly [4]. The chemical-curable resins have quite similar compositions like the light-cured one but have different initiators of polymerization. The initiator is per-compound: benzoyl peroxide, and it is combined with an aromatic tertiary amine [1]. Chemical cured composites have many disadvantages such as: color instability, problems with the proportions, mixing process, porosity, long curing time and short working time. However they also have some benefits like: they are easy to use, they do not require an additional curing equipment and they cure in places where the light cannot reach [1,7].

Some dental composites are dual cured. In these materials the polymerization starts after irradiation of light. They include photoinitiators like CQ and also iodonium salts and electron donors, which generate the reactive cationic species that start the polymerization process [3]. The aim of this double setting mechanism is to achieve a higher degree of conversion, especially at areas remote or hidden from the light source [5]. These composites are suitable to build the core of the tooth, when then crown is damaged and when the clinician will reinforce the tooth by luting prefabricated posts. The degree of conversion is different and depends on the depth of the cavity. In upper layers the polymerization is started by irradiation of light and deeper layers cured by chemical catalyst system [8].

The free radicals are generated upon irradiation of blue light. After this there is exchange of electron in initiator-co-initiator. Due to this process free radicals are produced through hydrogen abstraction. The initiator molecule becomes a ketyl radical while the co-initiator molecule becomes an amino alkyl radical. The remaining electron of the alkene group reaches the opposite terminal of the monomer and the whole molecule of the monomer becomes a radical. This molecule reacts with another monomer and it results in a chain reaction, which ends when two radicals react with each other. In this reaction some of monomers do not polymerize and they remain uncured. The relation between uncured monomers and cured resin is degree of conversion (DC) [2]. This is important parameter which influence on the physical and biological features of dental resin. Low conversion aggravates the biocompatibility of the material, because the unlinked molecules leak into surrounding tissues [9,10].

## 4. Photoinitiators

There are two types of photoinitiators: 1-type is trimethylbenzoyl-diphenylphosphine oxide (TPO) [11], benzoyl peroxide (BPO) and 2-type is camphorquinone (CQ), phenanthrenequinone (PQ), benzophenone (BP) [7] and 1-phenyl-1,2 propanodione (PPD) which combined two ways of polymerization [2]. This division is caused by different ways of production of free radicals by these photoinitiators, which are stated in detail in following parts of this review. The polymerization process can by initiated by α-cleavage (type-1 photosensitizer) and H-abstraction type (type-2 initiators). The photoinitiation system consists of photo-initiator and an electron donor or tertiary amine [2]. This photoinitiator system is stable in the presence of the oligomer at room temperature, as long as the composite is not exposed to light [4].

### 4.1. Type-1 Photoinitiators

**Figure 1:**
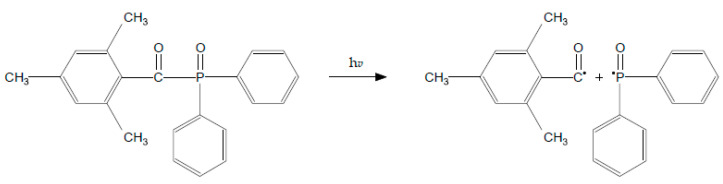
Alpha-cleavage of photoinitiation mechanism of trimethylbenzoyl-diphenylphosphine oxide (TPO).

Norrish type-1 photoinitiators improve material properties in dental resin composites. They have low energy bonds which after homolytic cleavage yields more active radicals and they allow photopolymerization by shorter wavelength, higher energy photon of violet light. Type-1 photosensitizers increase curing efficiency because of higher molar absorptivity. Another advantage of these initiators is improvement of tissue color matching as a result of low pigmentation due to shorter wavelength range absorption. The last profit is the reduction of elution of residual monomers that increase crosslink density of resin [12,13]. These photo-sensitizers do not require co-initiators and their color is not as yellow as photoinitiators type-2, but after polymerization they turn yellow due to high concentration of residual monomers [2]. Type-1 photoinitiators involves absorption of high energy violet light and subsequent excitation to singlet state and photochemical cleavage of carbon-phosphorus bonds. These photosensitizers undergo alpha-cleavage type of photoinitation mechanism, where the compound breaks down into two radicals (Figure 1). For example, trimethylbenzoyl-diphenylphosphine oxide undergoes rapid cleavage from triplet excited state and yields two radicals: trimethylbenzoyl radical and diphenylphosphinoyl radical. These radicals are able to initiate polymerization but with different rate constants [14]. The examples of 1-type photoinitors are TPO, bisacylphosphine oxide (BAPO) and monoacylphosphine oxide (MAPO) [2,13,15].

### 4.2. Type-2 Photoinitiators

**Figure 2:**
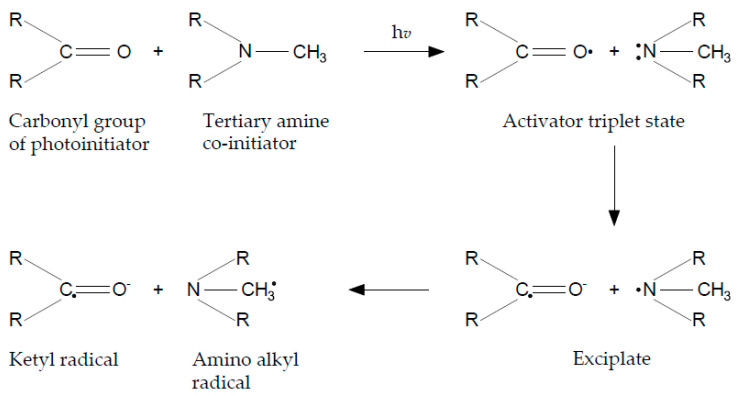
Photoinitiation by hydrogen abstraction (Type-2 photoinitiator).

The type-2 photoinitiator are e.g., CQ, PQ and BP with co-initiators, and their absorption band lies between 400–490 nm. The initiation is generally slower than photoinitiation caused by type-1 sensitizers, because is based on a bimolecular reaction [7]. The polymerization initiates by photons of visible blue light. The co-initiators of CQ are mostly aromatic tertiary amines. The concentration of CQ and co-initiators is obtained to gain a high degree of conversion. The polymerization is initiated by irradiation of blue light by the carbonyl group of CQ and transition into a triplet state via excitation into a singlet state (Figure 2) [2]. The radical formation is highly dependent upon the co-initiator type, the concentration and its structure [13]. This type photoinitiators are more useful than Norrish-type because of better optical absorption properties in the near visible wavelength region [16].

## 5. Photoinitiators for Resin Based Dental Composites

Benzophenone is a very common photoinitiator used in industry, especially for light-cured coatings, printing inks, paper production, board, metal coating to dry-film etc. (Table 1, 3) [7,17]. This photosensitizer is low-cost and efficient [18]. Benzophenone requires co-initiators to induce process of polymerization such as:s methyldiethanolamine, triethylamine, or ethyl 4-(dimethylamino) benzoate. Benzophenone is able to abstract hydrogen from alcohol, ether, alkyl amino, acid or thiol, but ketyl radical gained from the carbonyl compound is not able to start polymerization. It is caused by delocalization of unpaired electron and sterical hindrance [7]. Benzophenone has two broad bands of absorption spectrum: the first is weak and occurss at 320–370 nm and the second, stronger band, at 240–300 nm [18] with maximum of absorbance is 294 nm [7].

Some scientists are trying to combine BP with co-initiator in the one polymer chain. This incorporation has many advantages such as higher reactivity of photosensitizer, higher quantum yield of free radicals, faster reaction of polymerization [7,17]. Lee et al. in their studies use 4,4′-bis(*N*,*N*-diethylamino)benzophenone (DEABP) as a binary photosystem in light-cured resin for dental 3D printing. DEABP is a derivative from combining BP with 2-(*N*,*N*-dimethylamino)ethyl methacrylate (DMAEMA) and it is proved that this compound improves the degree of conversion and accelerates the velocity of polymerization. It can be also use as additive to CQ especially in 3D printing [19].

The most common photoinitiating system in dental composites is camphorquinone (Table 1, 4) and with co-initiators. It was invented by Dart and Nemcek in 1971 [15]. It is commercially used in e.g., Filtek Z250 (3M/ESPE Dental Product), Asteria Estelite (Tokuyama), Herculite XRV Ultra (Kerr Corporation) [6,19]. CQ is an alpha-diketone and it is type-2 photoinitiator. This photoinitiator absorbs visible light in the 360–510 nm wavelength range [5,20]. The absorbance maximum is at 468 nm [2,5,21,22], but other sources say that the absorbance maximum is 469 nm [13], 467 nm [23] and also 474 nm [2,5] These differences occur because CQ can be dissolved in various resin like TEGDMA or HEMA and it is called solvatochromic shift [2]. CQ is an intense-yellow-colored powder and it adds yellow tint to the uncured composite [24]. The color bleaches after irradiation [4], but Alvim et al. say that it has poor photobleaching and the yellow color remains the same after exposure to blue light [25]. This poor bleaching properties are caused by chromophore groups, which are components of CQ [13,25]. The yellowish staining may be a problem in color matching [12,26], so it led to less addition of photoinitiator and it changed the final properties of material. The staining is caused by co-initiator which undergoes oxidation with time promoting color change of the dental resin [27].

The concentration of CQ in dental resins vary from 0.17–1.03% in weight of the resinous portion [28]. However, Shintani et al. proved that the real range of CQ is 0.03–0.09 wt% of the dental composite [29]. These differences are caused by using various of amine co-initiators [30]. The composites with microparticle resins feature smaller amounts of CQ than conventional particles because of better light penetration [25]. The increase in CQ amount in dental resins leads to a higher degree of conversion and improvement of mechanical properties. Above the ideal level of CQ the degree of conversion does not increase [25]. The concentration of CQ should be optimal, otherwise it can compromise many properties such as: aesthetics, biocompatibility, biomechanical features and fillings can be susceptible to early wear. The aesthetic appeal of restoration can be impaired by residual unreacted CQ. These particles can also aggravate the overall biocompatibility, because they can leak into saliva and tissues of oral cavity. Insufficient concentration can also induce poor polymerization of dental composite and the mechanical properties will be weakened [30]. Alves et al. in their analysis proved that concentration of CQ influences: degree of conversion, mechanical properties and color features, but it does not affect the surface hardness. When the concentration of CQ is higher than 1 wt%, there was not significant different in surface hardness. Lower than 1 wt% concentrations of CQ reveal higher flexural strength [31].

The camphorquinone can generate free radicals by itself, but it is more efficient with incorporation of co-initiators. The emission spectrum of light source is critical to gain the efficient excitation of CQ molecules. The time to form triplet exciplex is limited, because the half-life of CQ triplet is ∼0.05 s. After this time the CQ triplet falls apart to basic state and the free radicals are not produced [30]. The efficiency of polymerization process depends on the steric structure of amine-derived radicals [28,29]. The most commonly used co-initiators are aromatic tertiary amines such as N, N-dimethyl-p-toluidine (DMPT), ethyl-4-(dimethylamino) benzoate (EDMAB) [13,29]. DMPT is reported to be toxic due to its relatively lower molecular mass. Another amine is EDMAB, which is also considered as cytotoxic, because it is not able to polymerize with monomers. Additionally, this amine promotes an increase intracellular formation of reactive oxygen species and rise of the intracellular glutathione, which can break integrity of cellular DNA [24,27]. The best biocompatible properties are displayed by 2-(*N*,*N*-dimethylamino)ethyl methacrylate (DMAEMA) and it does not leach out [2]. The aromatic amines such as EDMAB are more efficient than linear chain amines (DMAEMA), it is a very effective hydrogen donor [10], because of greater localization of electron through aromatic groups allowing electron transfer and reduces the possibility of back electron transfer. The back electron transfer retards hydrogen abstraction and eventually inhibits process of polymerization [13]. Schroeder et al. in their research proves that DMAEMA has lower activity than EDMAB, but it is the most biocompatible amine. When the concentration of CQ is 0.5 wt%, the degree of conversion of DMAEMA is 45% after 10 s light exposure and 62% when exposure lasts for 120 s. The effectiveness of DMAEMA is correlated with the concentration of CQ, but not with the time of exposure. When the concentration of CQ is 1.5 wt%, the degree of conversion is 76% after 10 s light exposure and 79% after 120 s light exposure. However, when the concentration of CQ is 0.5 wt%, the degree of conversion of EDMAB is 65% after 10 s exposure of light and 68% after 120 s after light exposure. When the concentration of CQ is higher and it is 1.5 wt%, degree of conversion is 76% after 10 s exposure and after 120 s light exposure degree of conversion is 79%. This shows that EDMAB is more effective amine despite the concentration of CQ and the time of light exposure [10]. Musanje et al. in their analysis reported that maximum hardness could be produced at concentration CQ:EDMAB 1.44:0.42 or 1.05:1.65 mol%. Degree of conversion was optimized at a CQ:EDMAB 2.40:0.83 mol%. Other conclusions are: the reduction of the CQ and EDMAB concentration below the optimal levels does not lower the contraction stress of dental resin and it is impossible to reduce contraction stress without influencing on Knoop hardness and degree of conversion [30].

Other co-initiators of CQ are: 2-ethyl-dimethyl benzoate, N-phenylglycine, p-octyloxy-phenyl-phenyl iodonium hexafluoroantimonate (OPPI) and diphenyliodonium salts (DPI) [2,12,32,33]. DPI was added to CQ/amine photoinitiator system to increase degree of conversion and rate of polymerization, and to reduce the back electron transfer process as well. DPI salts optimize the monomer conversion in two ways—through the reactivation of inactive free radicals and regenerating the photosensitizer to start polymerization process [33]. This co-initiator has many advantages during clinical application: first of all, the concentration of CQ and amines can be reduced, so the esthetic properties of composites will be better. Second, the efficiency of curing is increased and the dentist will save time during the application of restoration [12]. Adding to experimental resin composite bis-GMA/TEGDMA/CQ/EDAB different co-initiators (DPI or/and bis(4-methyl phenyl)iodonium hexafluorophosphate—BPI) makes material with properties (flexural strength and modulus) superior to the resin with a CQ-EDAB binary system, except for the final degree of conversion [34]. The addition of BPI/DPI increased the polymerization shrinkage strain of the experimental resins, as well as the rate of strain [34]. As new co-initiators in CQ based systems for the polymerization of methacrylates upon blue light irradiation can be introduced sulfinates and sulfonates (NapTS) [35]. The flexural strength and E-modulus obtained for the system CQ/NapTS were similar to the reference system CQ/EDB (e.g., 136MPa vs. 142MPa and E-Modulus: 9042MPa vs. 9240MPa), additional better bleaching properties and color stability of the final polymers with NapTS were noted [35].

CQ/amine photoinitiator system is the most common in commercial dental composites, however it has many disadvantages. Major disadvantage is yellow color of the restoration [2,21,23,31] and the camphorquinone maintains the same color after generation of free radicals [25]. Not only is CQ responsible for color of dental resin, the large amounts of tertiary amine may result in long-term darkening of these materials [22,23]. The CQ is consider as toxic [21,36], because it can change the metabolism of structural lipids which affects membrane integrity and permeability [2]. There are also reports that CQ have toxic effect on pulp cells and it is connected with the concentration of CQ in dental composite: the higher concentration, the stronger cytotoxic effect [31]. The CQ is less cytotoxic than BAPO, but it has genetic toxicity potential, due to production of ROS/RNS [37]. The other problem is that this system has two components and their interaction depends on the viscosity of the medium. In low-viscosity formulations the reduction of the triplet of CQ and amines is closely related to reaction of diffusion of these reagents. Whereas, in high-viscosity environment bimolecular systems are limited in their reactivity because the process is controlled by diffusion. To reduce this effect the amines are adjacent to photosensitizers by using polymerizable photoinitators and co-initiators [36]. It also produces an oxygen-inhibited layer [22]. These clinical problems have caused that other photoinitiators were considered for the production of commercial composites. Almeida at al. [27] evaluated the effect of different photoinitiator systems (based on CQ or BAPO or TPO) on the cytotoxicity, degree of conversion and the sorption and solubility behavior of a model adhesive resin containing different photoinitiation systems. Alternatively as initiators were added diphenyliodonium hexafluorophosphate (DPIHFP) with EDAB, BAPO, 1,3-benzodioxole (BDO), piperonyl alcohol (AP) and 1,3-diethyl-2-thiobarbituric acid (TBA). It was shown that experimental adhesive resins R_CQ+EDAB_, R_CQ+EDAB+DPIHFP_, R_BAPO_ and R_TPO_ showed similar degree of conversion values (higher than 60%), groups R_CQ+BDO_ and R_CQ+AP_ were the most cytotoxic materials [27].

Many scientists are trying to enhance the reactivity of CQ. Ulrich et al. in their studies proved that it is possible to increase reactivity of binary photosystem by covalently linking CQ with aromatic amines. They form covalently-bonded CQ amine photosensitizers from bromo precursors and cesium carbonate-catalyzed combining reaction with tertiary aromatic amines. It results in many new combinations of CQ and amines. The most reactive are systems gained from 10-bromocampherquinone, even at low concentration. The reactivity was measured by scanning photocalorimetry and then the degree of conversion was calculated. The degree of conversion of new compounds is in the range 55–65%. Another result is the compound 10-acetylcamohoroquinene reacts with ketone and ester group, but the stabilization of the ground state will be possibly gained after further investigations [36].

9,10-Phenanthrenequinone (Table 1, 5) is an alternative photoinitiator to CQ created in 1999. It is also a 2-type photoinitiator and it requires co-initiators like CQ. It is supposed to reduce yellow staining and to cooperate with CQ. This photoinitiator is an orange solid and it is aromatic diketone. The absorbance maximum of PQ is at 420 nm and it could be less yellow than CQ [33]. Albuquerque et al. [33] in their analysis compared influence of CQ and PQ on properties of resin-based composites. It showed that PQ has a higher relative photon absorption than CQ. The degree of conversion is the same for PQ and CQ regardless of the addition of a co-initiator. DPI in coaction with PQ increases the degree of conversion and also bring yellow values down. The materials containing CQ have a higher depth of cure than those with PQ. It is because of the absorption maximum of PQ, which is near the UV region and presents a curve extended to visible region of the spectrum and this decreases the light irradiance and reduces the penetration of light through restoration. The last feature which was compared was color. The resin-based composites including PQ have lower color stability than with CQ [33].

1-phenyl-1,2 propanedione (PPD) is a photosensitizer which forms free radicals by cleavage and by proton transfer from amine co-initiator (Table 1, 6) [5]. It is alpha-diketone and it has an aromatic group on one side of the carbonyl and a methyl group on the other [2,24]. This photosensitizer is a pale yellow viscous fluid and it ensures good compatibility with resins [5]. The range of absorbance is 300–400 nm and the absorbance maximum is 410 nm [2,24] and other sources say it is 393 nm [21] or 398 nm [12,32] and 400 nm [5]. CQ and PPD have almost the same light absorbance [38]. PPD can be used synergistically with CQ to increase the photopolymerization process [5,22]. It can be used alone or with a co-initiators such as tertiary amines or DPI salts [20]. PPD alone induced a degree of conversion mostly the same as CQ [14]. PPD is less yellow than CQ, which is desired feature in color matching, especially nowadays with the trend of bleaching. Another characteristic of PPD is an improvement of crosslinking by monomers in the network, which influences on kinetics of polymerization [20,32,34,35]. Park et al. examined the effect of synergistic usage of PPD and CQ. This analysis showed that PPD and CQ in ratios 1:1 and 1:4 achieved a maximum degree of conversion. It is caused by exploiting both methods of producing free radicals: photocleavage and proton abstraction. Another result is better absorption because of two different absorbance maxima: 468 nm for CQ and 410 nm for PPD. The connection of PPD and CQ allowed it to gain better esthetic properties: the hue of material shifted to less yellow shade [23]. However, Brandt et al. prove in their studies that correlation between PPD and CQ did not increase the degree of conversion of dental polymers and is dependent on the light emitter. When the lamp QTH XL 2500 is used the degree of conversion of CQ is 65.1%, DC of PPD is 58.8% and CQ/PPD is 61.4%. After exposure of LED UltraBlue IS the degree of conversion of CQ is 62.8%, DC of PPD is 61.6% and CQ/PPD is 60.9%. When UltraLume 5 is used the degree of conversion of CQ is 63%, DC of PPD is 62.9% and CQ/PPD is 62.6%. Using a quartz–tungsten–halogen (QTH) lamp causes that degree of conversion using PPD is the lowest. The most suitable light emitter for PPD is a light-emitting diode curing system, the conversion is the highest. When CQ and PPD are correlated the emitter of light does not have influence on the degree of conversion [9]. According to Brandt’s analysis the PPD reacts slower than CQ, but it does not reduce the degree of conversion and also PPD has a lower rate of polymerization [35,39]. The lower rate of polymerization can reduce crosslink density. The decreased crosslink density may result in softening of dental material in solvents and the material can be more vulnerable to enzymatic attack, which may manifest as an inferior biomechanical properties [40]. These results are depended on the type of light curing system. Using a halogen lamp the degree of conversion and hardness is worse than CQ. This is caused by the spectrum of light emission of halogen light and it is different than the absorbance range of PPD [41]. The Brandt’s studies from 2013 proves that PPD has sufficient properties to be used as a photoinitiator in dental resins [41].

2,4,6-trimethylbenzoyl-diphenylphosphine oxide (TPO) [10] is type-1 photoinitiator based on acylphosphine oxide (Table 1, 1). Commercially it is used in Tetric EvoCeram Bleach (Ivoclar/Vivadent) and Vit-1-escence (Ultradent Products Inc) [6,18]. It can be a stand-alone photoinitiator system or it can be used synergistically with CQ and it does not require co-initiators to accelerate photopolymerization process [18,31]. The use of Lucirin TPO eliminated the amine group and that increase stability of color upon aging [42] and the color stability is the highest according to resins containing BAPO and CQ/amines [38,42]. It is probably caused by higher molar extinction coefficient, which results in a greater consumption of molecules [43]. TPO has narrow wavelength absorption range 380–425 nm [44] and the maximum is 400 nm [44] or other source said it is 381 nm [26]. Due to shorter wavelength absorption range than CQ, the resin-based composites including TPO require new type of dental light curing units. The common light-curing units have spectrum limited to 420–490 nm and it is not sufficient for resin-based composites including TPO [10]. The best properties of TPO-material was gained when polywave light-curing units were used [6,36,45]. Single-peak light-curing unit has narrow range of light: 450–470 nm and the absorption of Lucirin TPO is out of this matter. However, polywave light-curing units are provided with extra light range: 400 to 415 nm and the exposure of resin containing TPO to the light is sufficient [46]. Additionally, Ilie et al. in their analysis proved that TPO can replace CQ, when the dual-wavelength LED units would initiate polymerization process [45]. The polymers including TPO can be polymerized in thick layers [18,31], it makes application of filling less time consuming, because the dentist does not have to put multiple thin layers of material into cavity. Unfortunately, thick layers increase polymerization shrinkage stress of material [19].

Because of the CQ is the most common photoinitiator in resin-based composites every property is compared to CQ’s feature in most analysis. The composites including TPO have higher degree of conversion than the composites containing CQ [18,36,46,47] The TPO-composites display an average 10% conversion increase. Miletic et al. [44] in their analysis proved that TPO is more efficient than CQ, because 0.86 wt% resulted in 74% degree of conversion and the lower concentration 0.22 wt% led to 68% DC. Another advantage of TPO-based material is faster polymerization than CQ-composites. The irradiation times are equal or greater than 3 s [48]. Lucirin TPO is more reactive than CQ even it does not require co-initiators. However, producer of Lucirin TPO says that using of amine component with TPO can decrease the inhibition of cure, which can be induced by oxygen [49]. The temperature increase of TPO resin-based composites is lower than CQ controls. The polymerization efficiency in dental resin is higher when TPO is used in dental resin, compared to CQ, PPD and BAPO’s efficiency, when the halogen light curing unit initiates the polymerization [46]. Lucirin TPO is more effective than CQ, because it produces two free radicals by α-cleavage, when CQ delivers only one. The first TPO’s radical is more competent as an initiator of polymerization, but it also can abstract protons from the medium and create a second radical [37,38]. The surface hardness is higher when TPO is used as a photosensitizer comparing to CQ and BAPO [50]. The flexural modulus and hardness were significantly higher in TPO-materials than CQ-composites, but flexural strength of TPO-composites and CQ-controls is similar. According to Popal et al. TPO and BAPO have lower cytotoxic effect on cell culture than CQ, and TPO is not genotoxic [50]. The first disadvantage of TPO-based composites is generation of higher polymerization stresses than CQ-controls [47]. The second disadvantage is lower depth of cure compared to CQ-containing mixture [6,37,46,51]. According to Van Der Lann et al. analysis TPO has no toxic effect on pulp [52]. For clinicians the most important advantage is esthetic aspect [48]. The TPO-based materials show great color stability [6,42,51,53] and also can mimic the optical characteristics of teeth such as color, opalescence and translucency [27].

Bisacylphosphine oxide (BAPO) is type-1 photosensitizer and it does not require a co-initiator to start polymerization process. Other name of BAPO is Irgacure 819 (Table 1, 2) [26]. It produces free radicals under alpha-cleavage type of photoinitiation mechanism. BAPO-containing resin is cured by violet light-emitting diode, the absorption range is 365–416 nm and the absorbance maximum is 400 nm [14], other source reports 371 nm [22]. It does not require tertiary amines [13,21]. BAPO is solid and has symmetric chemical structure and its solubility is poor in variety monomers and oligomers [54]. Ikemura et al. in their study showed that degrees of conversion of CQ-including resin and BAPO-containing resin are mostly the same [14]. However, recent articles proved that polymers containing BAPO have a higher degree of conversion, rate of polymerization, mechanical resistance [27]. Favarao et al. in their studies show that BAPO has the highest degree of conversion respect to TPO and CQ with different amine co-initiator [26,55]. Due to the absorption range it is important to choose proper curing lamp. BAPO-containing resins have higher degree of conversion when they are cured by high power LED light curing unit than by first-generation type LED unit [26]. Ullrich et al. in their studies proved that BAPO has increased reactivity compared to CQ/DMAB even in 16% of the standard photoinitiator concentration. The reactivity of BAPO is only limited by poor solubility [54]. When the CQ is used the top hardness (Knoop method) is 26.3 ± 0.7, the bottom hardness is 18.8 ± 1.2 and the depth of cure is 3.7 ± 0.1. When TPO is added the top hardness is 33.5 ± 2.4, the bottom hardness is 24.4 ± 1.9 and the depth of cure is 3.2 ± 0.1. Finally, when BAPO is used the top hardness is 32.9 ± 1.7, the bottom hardness is 25.6 ± 2.9 and the depth of cure is 3.6 ± 0.1. The surface hardness is higher when BAPO is a photosensitizer in dental resin comparing to CQ. The values of surface hardness TPO and BAPO are mostly similar. The depth of cure when the BAPO is used as the photosensitizer is mostly as the depth of CQ [50]. Another novel is that BAPO has the highest flexural strength according to TPO and CQ/amine [55]. The composite containing BAPO as a photosensitizer and UDMA as a matrix has shelf life problems, however this instability does not occur when other monomers are used [56]. BAPO a has weak genotoxic effect on cell culture [50]. The color of the polymer, when BAPO is used, does not turn into yellow [27].

**Figure 3:**
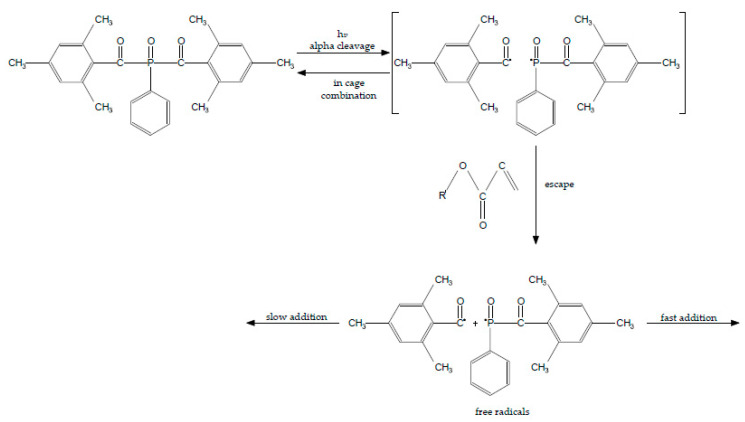
Alpha-cleavage of BAPO from triplet-excited state to yield radicals.

Type-1 photoinitiators undergo fast photolysis, generating benzoyl and phosphonyl radicals, which are very reactive and initiate the polymerization process (Figure 3) [14]. BAPO in its structure has two carbonyl groups; due to this feature it produces more free radicals than TPO. From one molecule of BAPO four reactive radicals could be generated and this is why BAPO is more efficient than TPO [38,43]. BAPO has a much higher molar extinction coefficient (870 L/mol cm) than that of CQ (33 L/mol cm). Additionally, BAPO has quantum yield five times higher than CQ [57]. BAPO, like others photosensitizer, can be synergistically used with CQ, but the efficiency of the production of free radicals has the highest value [17,25]. Lima et al. prove in their studies that BAPO does not require co-initiator. The values of flexural strength and elastic modulus of dental resin containing BAPO and DMAEMA (as co-initiator) are not higher and also the quantum yield did not increase, so adding a co-initiator to BAPO is unnecessary. However, using BAPO as a single photoinitiator causes values of flexural strength and elastic modulus much higher than using CQ and DMAEMA as initiators. [57]. According to Wang et al.’s analysis BAPO is very cytotoxic. The primary cells are very sensitive to BAPO and it significantly inhibits the growth of the cells [37]. Chiu in his analysis shows that BAPO in liquid form is as reactive as BAPO in its original solid form. They made a liquid mixture (LMBAPO) of bis-acylphosphine (BAP) and bis-acylphosphineoxideis (BAPO) in proportion 1:1. The absorption spectrum of LMBAPO is 340–400 nm, quite similar to BAPO. The biggest advantage of this liquid mixture is better solubility in most monomers and solvents. LMBAPO was used in ink industry, but it has not been used in dentistry yet [58].

## 6. Novel Photoinitiators 

In recent years new acylphosphine oxide photoinitiators were invented. They were namely, 9-(2,4,4,6-trimethylbenzoyl)-9-oxytho-9-phospha-fluoren (TMBOPF) (Table 1, 7) and 9-(p-toluyl)-9-oxytho-9-phosphafuluorene (TOPF) (Table 1, 8). These new photosensitizers have higher photopolymerization reactivity in visible and ultraviolet light. Their photopolymerization reactivity is comparable to BAPO’s. According to Ikemura et al. analysis the degree of yellow of thick photo-cured coating films initiated by TMBOPF and TOPF was reduced comparing to conventional BAPO [14].

**Figure 4:**
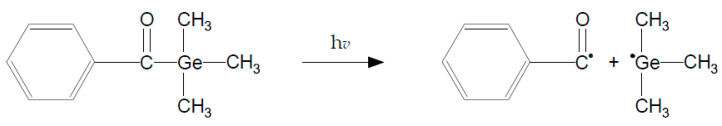
Alpha-cleavage of benzoyltrimethylgermane (BTMGe).

Benzoyl germanium substances—BTMGe (Table 1, 9) and DBDEGe (Table 1, 10) are novel visible light photoinitiators in dentistry. The photolysis of mono-germanyl-ketones in the cyclohexane solution was described 15–20 years ago. This process generates two radicals: benzoyl and germyl (Figure 4). Benzoyltrimethylgermane (BTMGe) is a yellow liquid and dibenzoyldiethylgermane (DBDEGe) is a yellow solid. The absorbance maximum of absorption of BTMGe is 411 nm and of DBDEGe is 418 nm.

These experimental photosensitizers are soluble in mono- and dimethacrylates. They are considered as low cytotoxic and they do not induce gene mutations. Moszner et al. [22] in their study showed that DBDEGe has significantly more intense absorption than CQ. The region of absorption of DBDEGe and BTMGe is close to CQ’s absorption spectrum, so these novel photoinitators do not require special light-curing unit with wide spectrum of light. Another feature of these germanium compounds is that they do not demand an amine co-initiator to start the photopolymerization process. DBDEGe has higher photocuring activity: setting time for DBDEGe is 3–5 s and for CQ is 8 s. The composites including the germanium photoiniatiator show only very slight yellowing. It was caused by the cross-linked organic polymer network. The yellowing can be reduce by adding a suitable UV-light stabilizer [22].

Ivocerin—dibenzoyl germanium is patented and is available only in select products from one manufacturer Vivadent (Table 1, 11). The absorption range of Ivocerin is 390–445 nm [25] and absorbance maximum is 418 nm [59]. Ivocerin like BTMGe and DBDGe forms at least two radicals, although the CQ-EMBO produce one aminoalkyl radical. Moszner et al. in their studies claim that dibenzoyl germanium can be relatively easy synthetized and it has the highest extinction coefficient comparing to other photoinitiators used in the analysis. They also proved that Ivocerin has low cytotoxicity and no mutagenic effect [60].

Recently, the photoinitiator based on dibenzoyl germanium has been added also to dental luting cements. Luting cements contain less amounts of filler, so the hardness cannot be compared to conventional dental composites. Luting cements containing dibenzoyl germanium (Variolink E, Ivocerin) are characterized by higher degree of conversion and color stability comparing to luting cements containing CQ. The degree of conversion of cement containing Ivocerin is about 87%, but cement containing CQ and tertiary amine has about a 44% degree of conversion. Alkhudhairy et al. also proved that Ivocerin-based cement has the highest Vickers micro-hardness and it is 47 VHN, additionally CQ-based cement has 33 VHN [60]. Delgado et al. prove in their studies that cements containing Ivocerin has flexural strength very similar to CQ-cements. The value of flexural strength of Ivocerin is 119.93 MPa and flexural strength of CQ is 120.41 MPa [61]. Ivocerin was also added to experimental radiopaque esthetic bulk-fill composites with aromatic monofunctional methacrylates [62].

Bouzrati-Zerelli at al. [63] analyzed a novel photoinitiating system (amine free) producing germyl radicals for the polymerization of representative methacrylate resins. The CQ/R3GeH/iodonium salt combination can be an effective photoinitiating system for the polymerization of methacrylate resins (Bis-GMA/TEGDMA or UDMA) in thin films or in thick composites upon exposure to a dental blue LED centered at 477 nm [63].

The coumarin-based iodonium hexafluoroantimonate (P3C-Sb) is alternative photoinitiator (Table 1, 12). It is a white colored powder used in the industry for cationic polymerization. Its absorption spectrum is near blue light and the maximum is 347 nm. P3C-Sb can be use alone, with tertiary amine or synergistically with CQ/amine photoinitiator system. The addition P3C-Sb to CQ and tertiary amines improves the degree of conversion and the kinetics of polymerization. Using P3C-SB and CQ or P3C-Sb with EDMAB did not achieve high values of degree of conversion and polymerization rates. There were no studies to analysis P3C-Sb as a single photoinitiator [64].

Wang at al. [37] proposed new photoinitiators [64] for bis-GMA/TEGDMA composite. Unfortunately the cytocompatibility of DTPs-photopolymerized BisGMA/TEGDMA polymers was inferior to CQ although their extracts exhibited low toxicity.

## 7. Summary

Nowadays in dentistry there are many different photoinitiator systems. The most common is the binary system camphoroquinone and tertiary amines and most dental light-curing units are created to suit to CQ’s absorption range. However, CQ has not been the best solution because of yellow shading caused by the yellow color of CQ and staining connecting with amines. The first type-2 photoinitator used in industry was BP. The CQ is more effective in dentistry, however BP is still used for example in 3D printing. Additionally BP with its co-initiators can be use with CQ and this combination improves the properties of the dental resin. Many scientists trying to link BP with amines and create new compounds. Maybe in the future the same process will be conducted without CQ. The invention of PQ gave new hope to change final color of the restoration. After analysis the properties of PQ-included composites it turned out that this photosensitizer is not sufficient, regarding stability of color of restoration after years in environment of oral cavity. The properties of resin-based composites improved after addition of PPD as a second photoinitiator. PPD cooperates with CQ and this mixture enhances the degree of conversion and the esthetic properties are satisfying.

The modern dentistry demands whiter hues of composites and request time-saving solutions. Now it is the turn of the Norrish photoinitators, which are acylphosphine oxide. They have a different type of polymerization, they do not require a co-initiator and they do not have a yellow tint. They also show greater stability of color during the years. Materials with Lucirin TPO as photoinitiator can be cured in thick layers more than the standard 2 mm. TPO enhances the degree of conversion comparing to CQ, the values can be higher up to 10%. Another feature of TPO is faster polymerization of TPO-containing composites that speeds up the application of the restoration. Unfortunately, TPO causes the bigger polymerization stress and has lower depth of curing. BAPO is not often used as a photoinitiator in dentistry but has many benefits. BAPO produces more free radicals than TPO, it is more photoreactive and that saves time of clinician during application of restoration. Regrettably, it has poor solubility in many monomers due to its chemical structure. Maybe the liquid mixture will increase usage of this photoinitiator but it has not been used in the dental field yet. The biggest disadvantage of type-1 photoinitiator is different absorption range (Table 2). These photoinitiator require different LED light-cured units. Manufactures should give the information about the photosensitizer used in their products and this is important to dentist to get to know the composition of material they want to use. Using unsuitable light-cured units can influence on values of degree of conversion, the mechanical properties and some parts of deeper layers can be uncured. All values of absorption ranges of photoinitiators are included in Table 1. The type-1 photosensitizers can be also used synergistically with CQ. This combination enhances the degree of conversion and reduces the yellowing after polymerization. It is important to use the polywaves light curing units which have wide spectrum of absorption.

During searching for the golden mean, many experimental photosensitizers have been discovered. The most promising are germanium compounds. They are soluble in most monomers and they have higher photocuring activity than CQ. Another benefit of BTMGe and DBDEGe is the absorption range, which is close to CQ and because of that they do not require different light-curing units. They also show slightly yellow color after polymerization. However, Ivocerin is a very promising photoinitiator. Ivocerin has great stability of color and its mechanical properties are better than CQ. Nowadays it is used in luting cement, but maybe in close future it will be used in flow and conventional composites. Next alternative photosensitizers which are able to change present commercial composites are novel acylphosphine oxide photoinitiators. TMBOPF and TOPF have higher photopolymerization reactivity, quite similar to BAPO, and they have proper stability of color. The last coumarin-based photoinitiators have a huge impact on esthetic properties of composites, but the maximum of absorption is at a much lower wavelength than standard light-curing units and it will demand changing the properties of dental equipment.

The branch of photoinitiators system is still developing. It is proven that this small part of composition of dental composites have huge influence on biomechanical and chemical properties of materials. However, it is an extensive problem to discover the golden mean which connects proper mechanical properties and esthetic appearance of restoration.

## Figures and Tables

**Table 1:** Chemical names, abbreviation, structural formula of photoinitiators used in review.

No.	Chemical Name	Abbreviation	Structural Formula	Type of Photoinitiator
1.	2,4,6—trimethylbenzoyl-diphenylphosphine oxide	TPO	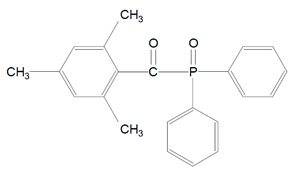	Type-1
2.	Bisacylphosphine oxide	BAPO	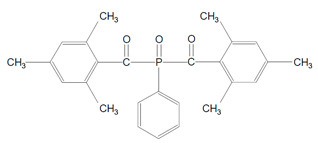	Type-1
3.	Benzophenone	BP	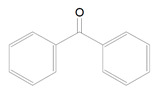	Type-2
4.	Camphorquinone	CQ	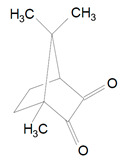	Type-2
5.	9,10-Phenanthrenequinone	PQ	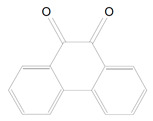	Type-2
6.	1-phenyl-1,2 propanedione	PPD	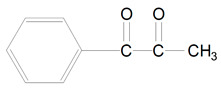	Type-2 and type-1
7.	9-(2,4,6-trimethylbenzoyl)-9-oxytho-9-phosphafuluorene	TMBOPF	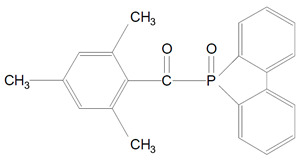	Based on germanium
8.	9-(p-toluyl)-9-oxytho-9-phosphafuluorene	TOPF	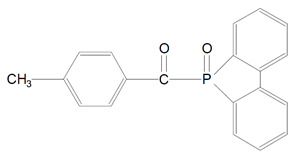	Based on germanium
9.	Benzoyltrimethylgermane	BTMGe	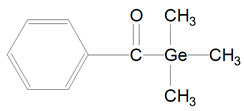	Based on germanium
10.	Dibenzoyldiethylgermane	DBDEGe	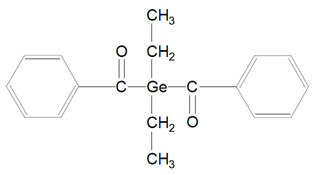	Based on germanium
11.	Ivocerin—dibenzoyl germanium	IVO	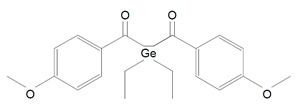	Based on germanium
12.	(7-ethoxy-4-methylcoumarin-3-yl) phenyliodo-nium hexafluoroantimonate	P3C-SB	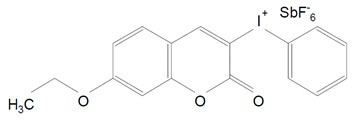	Based on coumarin

**Table 2:** Chemical names, absorption range [nm] and maximum of absorption [nm] of photoinitiators used in review.

No.	Chemical Name	Absorption Range [nm]	Absorbance Maximum [nm]
1.	2,4,6—trimethylbenzoyl-diphenylphosphine oxide	380–425	400
2.	Bisacylphosphine oxide	365–416	400
3.	Benzophenone	240–370	294
4.	Camphorquinone	360–510	468
5.	9,10-Phenanthrenequinone	No data	420
6.	1-phenyl-1,2 propanedione	300–400	410
7.	9-(2,4,6-trimethylbenzoyl)-9-oxytho- 9-phosphafuluorene	No data	No data
8.	9-(p-toluyl)-9-oxytho-9-phosphafuluorene	No data	No data
9.	Benzoyltrimethylgermane	No data	411
10.	Dibenzoyldiethylgermane	No data	418
11.	Dibenzoyl germanium (Ivocerin)	390–445	418
12.	(7-ethoxy-4-methylcoumarin-3-yl) phenyliodonium hexafluoroantimonate	No data	347

## Data Availability

The data presented in this study are available on request from the corresponding author
